# Autonomous Exercise Generator for Upper Extremity Rehabilitation: A Fuzzy-Logic-Based Approach

**DOI:** 10.3390/mi13060842

**Published:** 2022-05-28

**Authors:** Tanjulee Siddique, Raouf Fareh, Mahmoud Abdallah, Zaina Ahmed, Mohammad Habibur Rahman

**Affiliations:** 1 Department of Electrical and Electronics Engineering, University of Sharjah, Sharjah 27272, United Arab Emirates; u18105498@sharjah.ac.ae; 2 Department of Electrical Engineering, École de Technologie Supérieure, Montreal, QC H3C 1K3, Canada; mahmoud-a-y.abdallah.1@ens.etsmtl.ca; 3 Department of Physiotherapy, University of Sharjah, Sharjah 27272, United Arab Emirates; zzahur@sharjah.ac.ae; 4 Biomedical/Mechanical Engineering Department, University of Wisconsin-Milwaukee, Milwaukee, WI 53211, USA; rahmanmh@uwm.edu

**Keywords:** rehabilitation, decision-making system, fuzzy logic, range of motion, stroke

## Abstract

In this paper, an autonomous exercise generation system based of fuzzy logic approach is presented. This work attempts to close a gap in the design of a completely autonomous robotic rehabilitation system that can recommend exercises to patients based on their data, such as shoulder range of motion (ROM) and muscle strength, from a pre-set library of exercises. The input parameters are fed into a system that uses Mamdani-style fuzzy logic rules to process them. In medical applications, the rationale behind decision making is a sophisticated process that involves a certain amount of uncertainty and ambiguity. In this instance, a fuzzy-logic-based system emerges as a viable option for dealing with the uncertainty. The system’s rules have been reviewed by a therapist to ensure that it adheres to the relevant healthcare standards. Moreover, the system has been tested with a series of test data and the results obtained ensures the proposed idea’s feasibility.

## 1. Introduction

Stroke is the second or third leading cause of mortality in most countries around the world and is one of the major reasons for adult disability, affecting patients’ motor abilities and contributing to long-term neurological disabilities [1,2,3]. While mortality due to stroke has significantly decreased over the years, the long-term implications due to a stroke poses a great amount of impact on individuals and their family’s quality of life [4]. Loss of function on one side of the body, known as hemiparesis, is the most predominant disability caused by a stroke. This ultimately results in post-stroke patients being dependent on the people around them. This dependency is on the rise due to the imminent rise in the aging population. Although significant progress has been achieved in the medical management of stroke, most post-stroke care will continue to rely on rehabilitative therapies in the absence of a universally applicable or effective medical treatment [5]. Therefore, occupational and physical therapists have long been the foundation of post-stroke treatment. Unfortunately, due to financial constraints, the absence of adequate professional staff, and the labor-intensive nature of stroke rehabilitation, extensive therapy sessions for stroke patients are difficult to achieve [6].

While the term rehabilitation has a wide range of applications and is associated with various connotations around the world, the process can be broken down into a few key elements that are consistent across the board [7]. The process entails identifying a person’s problems and needs, linking the problems to relevant factors, formulating rehabilitation goals, planning and implementing measures, and finally evaluating the results. In short, the rehabilitation cycle consists of four steps: assessment, assignment, intervention, and evaluation [8].

Robot-assisted rehabilitation has been an active field of research providing therapeutic interventions through two prominent robotic structures, namely, end-effector-based robots and exoskeleton robots. End-effector-based robots are the ones where the upper or lower extremity is solely linked to the robotic device’s end-effector with a moveable distal handle [9]. As the rehabilitation process necessitates, this type of robot is defined by its adaptability to diverse sizes and types of movements [10]; however, the force generated at the distal contact affects the locations of other joints at the same time, making isolated joint movement challenging [11]. End-effectors have been in the scene for quite some time [12,13,14,15,16] showing promising results in the field of rehabilitation for the past two decades. Furthermore, new technologies continue to be adapted to these robots to improve their performance in replicating real-life therapy sessions [17,18,19,20,21]. Exoskeleton-type robots are the ones where the limb is attached to the end-effector in addition to multiple points along the robot. They are designed by encapsulating the limb with a splint or a bionic structure [9]. To control the movement of the limbs, the required torque for each joint is calculated. In contrast with end-effector robots, exoskeleton robots can be used in a more compact working environment. Exoskeleton robots have had similar success where multiple developments have shown great potential [22,23,24,25,26,27,28] and technological advancement only facilitates the improvement in the performance of these robots [29,30,31,32,33,34,35]. These robotic systems can easily give consistent training and monitor performance with great accuracy and reliability, and hence can provide crucial components for rehabilitation independently such as intensive movement therapy, just-right challenge, task-specific movement, and feedback on performance. Since intervention consists of specific repetitive methods, it is easier to automate the process, reducing the physical strain on therapists and effectively reducing the cost of therapy. Other elements of rehabilitation actively being made autonomous is the assessment and evaluation of the patient’s progress through various advanced biomechanical sensors. The use of Red Green Blue-Depth sensors, such as the Microsoft Kinect camera, inertial measurement sensors, and other sensors, have enabled the automated scoring of a portion of the Fugl–Meyer Assessment Scale [36]. The wide range of assessment scales and methodologies (sensor-based, tracking systems, computer-based, and so on) make automating assessment a particularly promising avenue of research [37].

Evidently, the only step of rehabilitation that is yet to be automated and is still fully reliant on therapists is the assignment of treatment. Few works have been performed in this area, such as a traditional computerized exercise expert system [38], which was designed to create personalized exercise prescriptions for elderly individuals with the help of a panel of experts in medicine, exercise physiology, health promotion, exercise psychology, and gerontology. More recently, an autonomous system [39] was created for pediatric rehabilitation where the system provides a user interface through which the expert can configure the session execution parameters. Depending on the parameters, the Java-based Simple Hierarchical Order Planner completes the planning process to produce a valid plan with exercises for each session. To the best of the authors’ knowledge, no commercial systems exist that can automatically generate a complete rehabilitation strategy from the initial functional assessment data for stroke patients, so therapists must still properly identify the patient’s problems through a reliable diagnosis and the appropriate clinical measures to assess the treatment’s effectiveness.

There have been a substantial amount of databases with information on each patient since the introduction of Electrical Medical Records (EMRs). These databases store information from previous hospital visits, diagnoses and interventions, lab results, medical scans, and clinical narratives that can be used to assist clinicians with diagnoses and treatment decision-making [40]. The rationale behind the decision-making process in medical applications is a complicated process that encompasses a certain amount of uncertainty and ambiguity [41]. Fuzzy logic systems excel in dealing with ambiguous and imprecise data, which make them a common application for medical diagnosis systems. Intelligent systems employ various technologies including expert systems, artificial neural networks [42], fuzzy systems [43], and evolutionary computation. Each technique has its perks depending on the application, while the combination of two or more techniques inevitably improves systems’ adaptability, robustness, fault tolerance, as well as speed [44].

In this paper, an exercise generation system for Upper Extremity (UE) stroke rehabilitation is developed using a fuzzy-logic-based model. The main contribution of the paper is to develop an autonomous system that generates a treatment plan in the form of rehabilitative exercises depending on some input parameters. These input parameters consist of the different physical characteristics of the patients concerned with stroke, which in our case are the joint range of motion (ROM) and the muscle strength. A library of active range of motion (AROM) exercises was used and divided into three subsequent progress levels. Exercise levels were chosen for the patient, along with the amount of stretching and strengthening exercises they require based on rules that were defined with the help of a therapist. The system was broken down into a simple fuzzy tree structure with two fuzzy inference system (FIS) blocks utilizing the Mamdani approach [45]. The input to the first FIS was the shoulder ROM data, i.e., the shoulder flexion, abduction, and external and internal rotation. While the input to the second FIS was the strength data of the muscle of interest, and the output from the first FIS, which was the level of exercise. There are three outputs in the system, the level of ROM exercises, which is the output of the first system, determined based on ROM data. The intensity of the strengthening exercises based on the previous input and strength data is an output of the second FIS, and finally, the intensity of the stretching exercises depending on both the aforementioned parameters is also an output of the second FIS. These outputs can help in selecting the exercise plan that is appropriate for the patient. We also defined a strength scale that was employed in the system. It was designed to make the system more compatible with other autonomous systems, providing sessions without the need for a therapist’s supervision. The best reference to compare to in physiological recovery is usually to oneself. Since stroke affects patients by reducing function only on one side of their body while often leaving the other side unharmed, we can assume that the maximum strength that a patient’s muscle should have is as high as their healthy side’s muscle; therefore, we proposed to collect the maximum voluntary contraction (MVC) data of the patients’ deficit muscles and define it as a percentage of the MVC on the healthy side. The scale was defined with six levels with the aforementioned therapist.

The outline of this paper is as follows: Section 2 discusses the technical background of the paper, which includes a brief explanation of the medical knowledge that needs to be considered. Section 3 covers the structure of the proposed decision-making system and its working principle, while Section 4 discusses the results obtained. Finally, concluding remarks as well as future works appear in Section 5.

## 2. Design Requirements and Background

While building a system that deals with decision making, it is crucial to understand the parameters going into said system to ensure that they are utilized in an efficient as well as safe manner throughout the procedure. In this section, common patient parameters that are examined in stroke cases will be explored.

### 2.1. Range of Motion

The term range of motion refers to the measurement of how far a person can move a specific joint or muscle. How far a joint can be moved may be limited due to several factors, such as the presence of spasticity, weakness of the muscle, etc. By measuring the ROM of joints, the effect or requirement of therapeutic measures may be determined. The functional ROM [46] of the UE was divided into three ranges with the help of a therapist; low range, medium range, and advanced range—this can be seen in Table 1, Table 2 and Table 3.

### 2.2. Muscle Strength

Maximum voluntary isometric contraction (MVIC) is an essential method for measuring and evaluating muscular strength, which has high levels of reliability. Furthermore, in many experiments, MVIC may be utilized to replace the normalization of electromyography (EMG) data, which are used to assess muscle conditions. As a result, MVIC has established a gold standard in patient evaluation and muscle activity research [47]. For the purpose of this paper, we have designed a muscle strength evaluation criterion with the help of an expert therapist. The strength of the muscle is defined as a percentage of the healthy side, i.e., the MVC or MVIC of both sides are collected, and the paretic side’s strength is calculated as a percentage of the unaffected side. This is then divided into subsequent ranges to be used in our inference system as can be seen in Table 4. Studies show that practicing at a minimum intensity of 60% 1-Rep Maximum and a maximum of 12 repetitions per set can help hemiparetic people develop their strength [48]. So we propose to give patients resistive loads weighing at 60% of their maximum strength to increase their strength and reduce muscle atrophy.

### 2.3. Spasticity

Spasticity can be defined as the velocity-dependent hyper-excitability of stretching muscles, characterized by enhanced tendon reflexes, increased resistance to passive movement, and neuronal inhibition in control. UE spasticity is one of the most serious effects of stroke, which can cause considerable functional disability by limiting the ROM or slowing the pace of limb movement [49,50]. Studies suggest that stretching exercises contribute to reducing spasticity in post-stroke patients; therefore, we have proposed to include stretching exercises of two levels depending on the patients’ ROM as well as their strength.

## 3. Methods and Materials of the Fuzzy-Based Decision-Making Scheme

**Figure 1:**
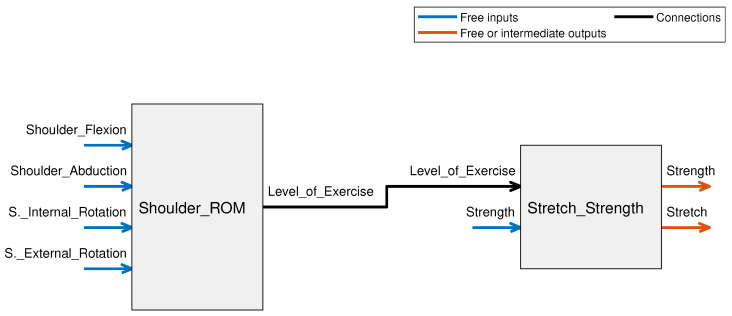
Block diagram for overall FIS illustrating the fuzzy tree structure where the output of the Shoulder_ROM FIS block (Level_of_Exercise) is input into the Stretch_Strength system.

Rule evaluation is one of the most critical aspects of designing a functional fuzzy-logic-based system. The number of rules in the system is determined depending on the number of input parameters and their corresponding membership functions. While it is possible to create a FIS with a large number of inputs, this often leads to a very high number of rules, which may become difficult to keep track of; therefore, we have broken the system down into a fuzzy tree structure with two FIS blocks as shown in Figure 1. The first system determines the level of ROM exercises the patient can be assigned. This is determined using the different ROMs of the shoulder. The second system determines the intensity of stretching and strengthening exercises the patient will be capable of carrying out. The output of the first system is fed into the second system as one of the inputs (indicated by the black arrow in the figure). The strength input and output both have the same membership functions, however, the output strength is applied depending on both the input strength as well as the patient’s shoulder ROM indicated by the “Level_of_Exercise” output from the shoulder ROM fuzzy system. This is discussed further in Section 3.2.2.

**Figure 2:**
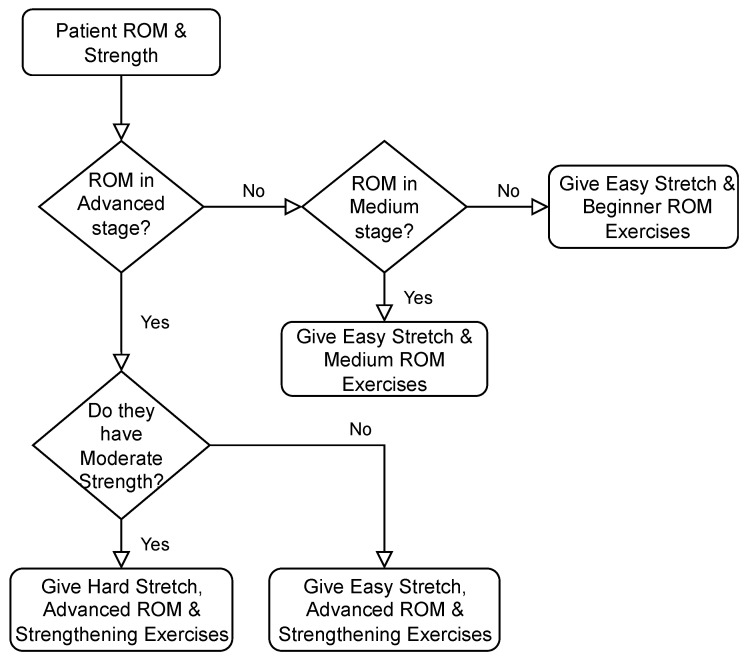
Decision Flowchart for Rule Evaluation.

The decision-making process that was used is as follows: If the patient’s ROM is in the advanced stage, give them strengthening exercises and advanced level ROM exercises. If not, give the corresponding level of exercise with no strengthening exercises. If strength is above moderate, give hard stretching exercises, otherwise, give easy stretching exercises. The process can be seen more clearly in Figure 2.

**Figure 3:**
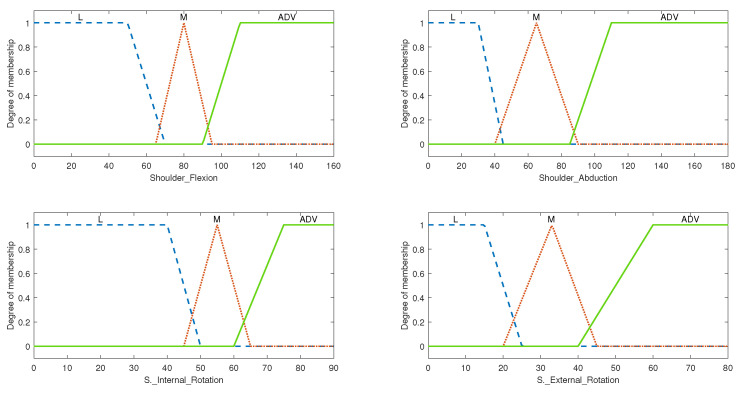
The membership function plot for level of exercise input, where L represents low ROM, M represents medium ROM, and ADV represents advanced ROM.

The system was designed based on fuzzy input and output triangular and trapezoidal membership functions. The triangular membership functions are given in Equations (1) and (2), where *x* corresponds to the input value of the system, and *a*, *b*, and *c*, corresponding to the start point, midpoint, and endpoint of each triangle, respectively. Equation (2) is a compact version of Equation (1), which gives a clearer explanation of the function parameters. The other type of membership function that was used was the trapezoidal type shown in Equation (3). The same principle applies here, except the midpoint is now represented by two points instead of one, which is useful in cases where the membership plateaus at a point instead of peaking. Parameters *b* and *c* define the shoulders of the membership function, and *a* and *d* define its feet. This case can be seen in both the “L” and “ADV” memberships of the level of exercise input seen in Figure 3. The values of the parameters are set by the designer of the system according to their understanding of the applicable memberships. All the rules used the AND function, which used the minimum method, and the defuzzification method that was chosen was centroid. This paper takes as an example the shoulder, however, the FIS for the elbow and wrist can easily be defined following the same framework that was applied to the shoulder to produce a complete UE personalized exercise generation system.

(1) $$f\left(x;a,b,c\right)=\left\{\begin{array}{cc} 0, & x\leq a\\ \frac{x-a}{b-a}, & a\leq x\leq b\\ \frac{c-x}{c-b}, & b\leq x\leq c\\ 0, & c\leq x \end{array}\right\}$$

(2) $$f\left(x;a,b,c\right)=\max(\min\left(\frac{x-a}{b-a},\frac{c-x}{c-b}\right),0)$$

(3) $$f\left(x;a,b,c,d\right)=\max(\min\left(\frac{x-a}{b-a},1,\frac{d-x}{d-c}\right),0)$$

### 3.1. Shoulder ROM FIS

This system can be defined in terms of three main subcategories, the input variables, the output variables, and the rule evaluation performed. It consists of 4 input variables that make up the ROM of the shoulder region and an output variable that determines the overall level of ROM exercises to be assigned to the patient. The output is later fed into the second subsystem that deals with strength and stretch variables.

#### 3.1.1. System Variables

The input of this FIS consisted of 5 input variables with 3 membership functions each: shoulder flexion, shoulder extension, shoulder abduction, shoulder internal rotation, and shoulder external rotation. While evaluating the rules, it was found that none of the exercises in the composed exercise library directly affected or were affected by the shoulder extension range; therefore, we were able to ignore that input, decreasing the number of inputs from 5 to 4. Given that each input has 3 membership functions, the product of each of the input membership functions resulted in a total of 81 rules. With the inclusion of the shoulder extension input, the system would then have 243 rules, which is significantly higher. The fuzzified inputs with their membership functions are shown in Figure 3. The medium range was defined with a triangular type membership function while the low and advanced ranges were defined with a membership function of the trapezoidal type. The dashed plot colored in blue classifies the patient’s input ROM as “Low”, the dotted red-colored plot classifies the input ROM as “Medium”, and the solid green-colored plot classifies it as “Advanced”. These membership plots were defined by closely following the ranges discussed in Table 1.

**Figure 4:**
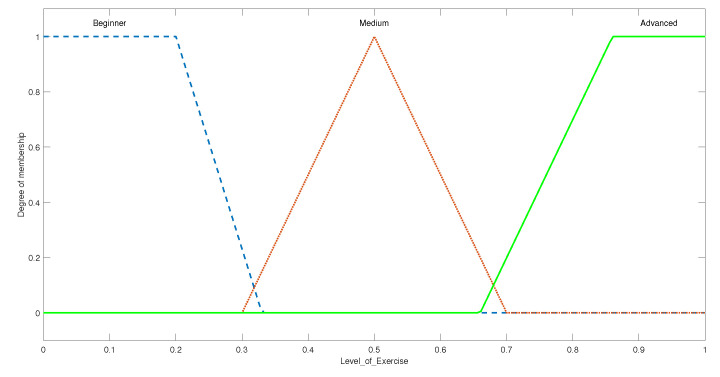
The membership function plot for level of exercise output.

This FIS was designed to determine the level of ROM exercise that must be given to the patient where the levels are defined as beginner, medium, and advanced. The output variable with its membership functions can be seen in Figure 4. The universe of discourse for the output of this FIS is defined over a range of 0 to 1; where an output of 0.5 translates to a medium level of exercise, less than 0.5 translates to a beginner-level exercise, while more than 0.5 translates to an advanced level of exercise. The therapist may assign the exercises according to the aforementioned rules.

#### 3.1.2. Rule Evaluation and Overall Setup for Shoulder ROM FIS

**Figure 5:**
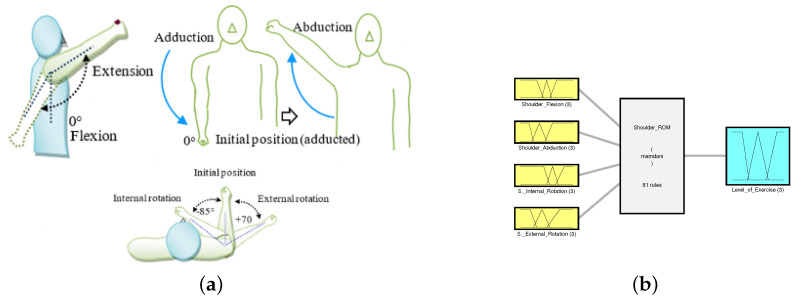
Shoulder ROM FIS and its respective motions: (**a**) Schematic of shoulder joint motions; (**b**) the FIS for determining the level of exercise.

The total number of rule definitions in this system added up to a total of 81. A comprehensive library of exercises retrieved from [51] was divided into three main levels: “Beginner”, “Medium”, and “Advanced”, matching the output of the FIS as seen in Figure 5b. The corresponding movements can be seen in Figure 5a to obtain a clear idea of the motions. Beginner exercises included the general single joint motions such as the shoulder vertical and horizontal flexion/extension, abduction/adduction, and internal/external rotation. The medium levels were comprised of multi-joint exercises that required a higher ROM, followed by the advanced level exercises which replicated everyday functional tasks. The rules were evaluated specific to this exercise library in order to achieve the most accurate outcomes. They were evaluated such that, the flexion and abduction ranges of the shoulder holds the highest weight in determining whether the patient can progress to medium level exercises. This is performed because all medium-level exercises require a good range in the flexion and abduction motion; however, when moving to the advanced level exercises, all types of motions are thoroughly utilized and therefore all inputs hold quite a high weight, resulting in very few combinations where the advanced level exercises were assigned. This ensures a more critical evaluation and assignment of exercises; ideal for cases with stroke patients as they are weak, and recovery is often more challenging as compared with other cases.

### 3.2. Stretch and Strength FIS

This subsystem deals with the patient’s strength and level of ROM exercise as inputs, and assigns the correct capacity of strengthening and stretching exercises. The rule evaluation performed in this part is analogous to the decision-making scheme presented in Figure 2.

#### 3.2.1. System Variables

**Figure 6:**
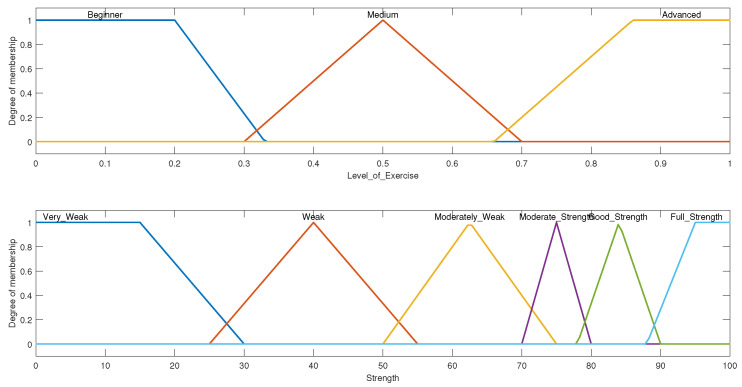
The input membership functions for strength/stretch FIS.

The input variables in this fuzzy system include the level of exercises determined from the previous FIS- with the same three membership functions, and the percentage of the strength of a muscle as compared with its unaffected counterpart. The second input was divided into 6 membership functions following the previously defined ranges in Table 4. The membership function for the input strength variable can be seen in Figure 6.

**Figure 7:**
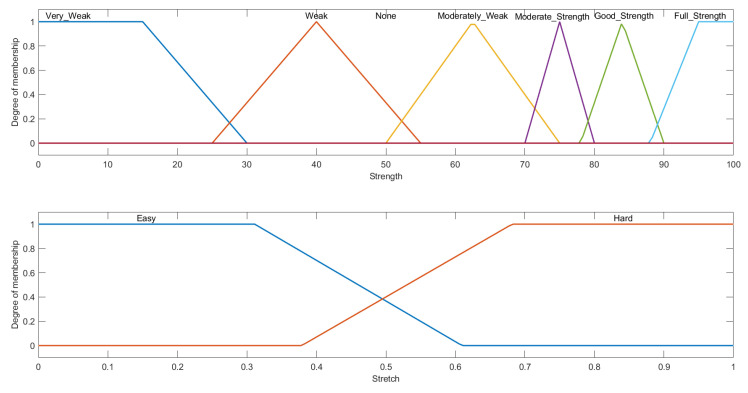
The output membership functions for strength/stretch FIS.

This FIS consisted of two output variables. One was the amount of strength of the exercise to be assigned, which was designed exactly like the input strength and the level of stretch. The level of stretch was divided into two trapezoidal membership functions: easy and hard as shown in Figure 7.

#### 3.2.2. Rule Evaluation and Overall Setup for Stretch and Strength FIS

After determining the level of ROM exercise, we designed the second FIS to determine whether the patient should be given strengthening exercises, and if so, at what capacity. Along with that, stretching exercises were included as it is one of the commonly used methods of intervention used to tackle spasticity challenges in post-stroke patients. The two inputs, level of exercise and percentage of strength, have three and six membership functions, respectively, the product of each of the input membership functions resulted in a total of 18 rules; therefore, the system consisted of a total of 18 rules. They were designed so that strengthening exercises are only assigned when the patient has regained a considerable amount of their ROM (i.e., the level of exercises assigned was in the advanced range). When the range was at the advanced level, strengthening exercises were a direct mapping of the input strength. As for stretching exercises, easy stretching is assigned for patients with beginner and medium ROM as well as for a few of the advanced ROM. When patients reach moderate to full strength, hard stretching exercises are prescribed. It is worth noting that most ROM exercises are transformed into strengthening exercises by adding a resistive element to them. To build strength and reduce muscular atrophy, the therapist can assign strengthening exercises to the patients with resistive loads weighing 60% of the strengthening capacity assigned by the system as discussed in Section 2.2 and can be seen more clearly in Equation (4). For example, if a patient were to have a strength of 70%, and their 1-Rep maximum on the healthy side was 2 pounds, then the therapist should assign strengthening exercises with the resistive load weighing 0.84 pounds to improve their strength. For a patient that has 90% strength in their teres minor and infraspinatus muscles, with their 1-Rep maximum at 2 pounds, they would be assigned strengthening exercises with a resistive load of 1.08 pounds, i.e., advanced level ROM exercises with resistive loads. At the same time, hard stretching exercises must be assigned such as the teres minor stretch. If the patient has low strength, then light stretching exercises such as sleeper stretch can be assigned [48].

(4) ResisitiveLoad=1RM×PercentageStrength×60%

where 1*RM* is the 1-Rep maximum and the percentage strength is the strength of the impaired muscle.

## 4. Results and Discussion

The proposed fuzzy-logic-based exercise generation system was tested with a set of input variables that included typical patient biomechanical data, including shoulder joint ROM and strength (represented by the percentage of MVC). The shoulder ROM data, which included shoulder flexion, abduction, and external and internal rotation, were fed into the first FIS. The strength data of the muscle of interest were the input to the second FIS, while the output from the first FIS was the level of exercise. The system has three outputs, the first of which is the level of ROM exercises, which is decided based on ROM data. The second FIS outputs the intensity of the strengthening exercises based on the prior input and strength data, and finally, the intensity of the stretching exercises are determined based on both of the aforementioned parameters. The tested input data and results of the first and second FISs are shown in Table 5 and Table 6, respectively. For the first system (shoulder ROM FIS), in cases 1 through 4, since shoulder flexion and abduction are in low ranges alternatively, a beginner exercise level (<0.5) is assigned by the system. In case 2, although both shoulder internal and external rotations are in the medium range, as can be seen in Table 1, the exercise level does not advance much, i.e., the level of exercise increases from 0.1326 to a mere 0.1529. In case 3, all angles are in the medium range except for abduction, despite this fact, the resulting output (0.1503) does not increase. Meanwhile, we can see in case 4 that all input ranges except shoulder flexion are in medium range with the resulting output level of exercise (0.3255) higher than the earlier cases i.e., less than 0.16. This brings us to the conclusion that the exercise level can advance from beginner to medium only when both flexion and abduction are in the medium range, and abduction has a higher weight in this decision than flexion. This matches the rules defined for the system, as medium-level exercises in our chosen exercise library require a higher abduction range compared to the rest. This is further confirmed in case 6 where low ROMs for internal/external rotation still resulted in a medium exercise level (0.5) assignment since the other two ROMs were in the medium range. In case 7, however, shoulder external rotation being in the medium range caused the output (0.5) to remain in the medium range despite having advanced ROM in other motions. This too is analogous to the rules defined in the system, as advanced-level exercises required advanced range in the external rotation for the exercise library used. The following three cases have an advanced range in most motions and therefore output a value higher than 0.5, which represents an advanced level of exercise. In the eleventh run, the shoulder internal rotation value is set to be 100 degrees, but the range for that input is defined over 0 to 90 degrees; therefore, no rules are fired for this combination, and the default value of the medium range is set although all input angles are in the advanced range. For cases 12 to 14, the ROMs are mostly in advanced range with the exception of one of the motions in each case, which is set to a low ROM. This results in the assignment of beginner-level exercises (<0.5). In the final case, all the motions are in advanced range except for flexion, set with a medium ROM, the resulting output remains at the medium level of exercise.

For the second system (stretch and strength FIS), the resulting outputs are shown in Table 6. Since the level of exercise determined by the system from cases 1 through 6 all fall under the “low” and “medium” level, we see that the prescribed strength intensity is zero. This is because the system must not assign strengthening exercises if the patient cannot perform advanced level ROM exercises. The type of stretch assigned is ”Easy” for all the cases where a low or medium level of exercises are assigned, apart from the case with medium range with 90% strength, where “Hard” stretching exercises are prescribed. This is consistent with the desired outcome since, if a patient has a substantial amount of strength, tightness of their muscle would be the other viable reason for restricted ROM. The following four testing values all fall under advanced ROM and therefore we start seeing the assignment of strengthening exercises. For the 11th case, the ROM is set to the medium range instead of advanced due to incorrect range input for the internal rotation ROM, therefore, strengthening exercises are not assigned. In the cases from 12 to 15, the level of exercises assigned are in the beginner and medium range, so strengthening exercises are not assigned.

**Figure 8:**
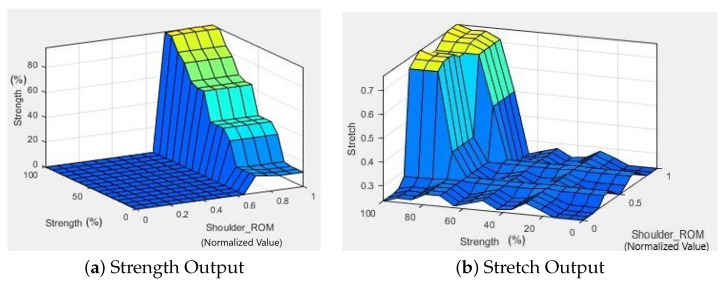
Surface Viewer plot of the second FIS. (**a**) Strength output plot shows that the strengthening exercises are only assigned when the input shoulder ROM (level of exercise) is higher than 0.5, which implies an advanced range, while the intensity of strengthening exercise (output) rises steadily with increased input strength. (**b**) Stretch output plot shows that a high shoulder ROM, i.e., level of exercise > 0.5, with high input strength results in “Hard Stretching” output, represented by a value greater than 0.5, and for lower ROM and strength, “Easy Stretching” output, represented by a value less than 0.5.

The surface viewer plot depicts the output surface for a system output against one or two system inputs. Against one input, a 2D plot is produced, while against two inputs, a 3D plot is produced; however, the number of variable inputs cannot exceed two. In the case of four inputs and one output, as computer monitors are not designed to display five-dimensional structures, the Surface Viewer creates a three-dimensional output surface with any two of the inputs varying, while the other two are kept constant. In Figure 8a, the output is displayed on the z-axis as a percentage of strengthening exercise assigned, the inputs are displayed on the x and y-axes where the shoulder ROM ranging from 0 to 1 signifying the levels is displayed on the x-axis and the percentage of input strength is displayed on the y-axis. It is evident that the strengthening exercises are only assigned when the input shoulder ROM is higher than 0.5, which implies an advanced range, while the intensity of strengthening exercise (output) rises steadily with increased input strength. For Figure 8b, the inputs remain the same, but the output is the intensity of the stretching exercises, i.e., Easy (<0.5) and Hard (>0.5). This surface viewer plot is a little more difficult to decipher, but in general, when the input ROM is in the medium stage, a higher input strength value (good strength) is required to move from easy stretch to hard stretch. While, when the input ROM is in the advanced range, moderate input strength is sufficient to move from easy stretch to hard stretch. The system was tested with a series of test data and the results obtained ensures the proposed idea’s feasibility. From the results presented in Table 5 and Table 6, and Figure 8, we can conclude that the proposed FIS can determine the appropriate therapeutic intervention in terms of exercises, based on patients ROM and strength data. The proposed method will help therapists to input patient data, and receive the appropriate levels of exercises that they must assign to the patient. Currently, there exists no such system for stroke rehabilitation, leaving manual therapy assignments by therapists as the only other option. By automating the assignment of exercises, this system can easily be integrated with another robot-assisted system to provide increased autonomy and less dependency on therapists; however, the system is highly dependent on the chosen exercise library and its consequent level divisions. Additionally, it is known that therapeutic interventions are also influenced by the type of stroke (ischemic or hemorrhagic) and the anatomical location of the afflicted area of the brain. These were not considered as input variables of the system due to the complex relations between these input variables and the corresponding therapeutic intervention.

## 5. Conclusions

This paper details the design and structure of a fuzzy-logic-based system that determines the correct exercise prescription based on patient data, i.e., joint ROM and muscle strength. First, the correct reference ROM was defined for a joint with the help of a therapist. The ranges were later divided into three levels: low, medium, and advanced. Our goal was to create a system that can work in conjunction with other autonomous systems without therapists’ supervision, so we needed to define a scale that could be used without evaluation by a therapist. We can assume that the maximum strength that a patient’s muscle has is comparable to their healthy side; therefore, the MVC data of the patient’s deficit side are defined as a percentage of the MVC on the unaffected side. To reduce the number of input variables going into the FIS, we broke the system down into two parts of a fuzzy tree. The first part determines the level of AROM exercise assigned, while depending on that value, along with the strength data, a decision is made about whether strengthening exercises and stretching exercises will be given or not, and at what capacity. Moreover, to generate a more personalized treatment plan, a neuro-fuzzy approach can be followed. As previously discussed, a combination of AI techniques greatly increases the performance of decision-making systems and therefore our next goal is to build a system employing these techniques.

## Figures and Tables

**Table 1:** Shoulder Range Division.

	Low Range	Medium Range	Advanced Range
	(Degrees)	(Degrees)	(Degrees)
S. Extension	ROM < 30	30 < ROM < 38	38 < ROM < 50
S. Flexion	ROM < 70	70 < ROM < 92	92 < ROM < 110
S. Abduction	ROM < 45	45 < ROM < 90	90 < ROM < 110
S. Medial Rotation	ROM < 45	45 < ROM < 64	64 < ROM < 70
S. Lateral Rotation	ROM < 20	20 < ROM < 40	40 < ROM < 60

**Table 2:** Elbow Range Division.

	Low Range	Medium Range	Advanced Range
	(Degrees)	(Degrees)	(Degrees)
E. Flexion	ROM < 110	110 < ROM < 124	124 < ROM < 135
E. Extension	ROM > 10	10 > ROM > 5	5 > ROM > 0
Supination	ROM < 18	18 < ROM < 30	30 < ROM < 50
Pronation	ROM < 13	13 < ROM < 30	30 < ROM < 50

**Table 3:** Wrist Range Division.

	Low Range	Medium Range	Advanced Range
	(Degrees)	(Degrees)	(Degrees)
W. Flexion	ROM < 20	20 < ROM < 30	30 < ROM < 50
W. Extension	ROM < 30	30 < ROM < 40	40 < ROM < 60
U. deviation	ROM < 15	15 < ROM < 19	19 < ROM < 25
R. deviation	ROM < 10	10 < ROM < 18	18 < ROM < 20

**Table 4:** The defined strength scale.

Linguistic Range	Strength Range in Percentage
Very Weak	0–25%
Weak	25–50%
Moderately Weak	50–70%
Moderate Strength	70–80%
Good Strength	80–90%
Full Strength	90–100%

**Table 5:** Presents the results of the first system, shoulder ROM FIS with shoulder joint motions as inputs and the resulting level of exercise as output.

	Shoulder Flexion ROM	Shoulder Abduction ROM	Shoulder Internal Rotation ROM	Shoulder External Rotation ROM	Level of Exercise (Output)
Case 1	38	29	35	10	0.1326
Case 2	55	40	47	22	0.1529
Case 3	71	34	50	25	0.1503
Case 4	55	47	51	23	0.3255
Case 5	88	59	62	37	0.5000
Case 6	73	46	40	15	0.5000
Case 7	93	78	70	41	0.5000
Case 8	95	88	81	58	0.6920
Case 9	97	91	85	61	0.8443
Case 10	108	108	90	75	0.8722
Case 11	130	180	100	80	0.5000
Case 12	100	95	40	15	0.1498
Case 13	60	100	65	50	0.1518
Case 14	105	40	68	53	0.1518
Case 15	85	105	70	55	0.5000

**S. Flexion ROM:** Low < 70°, Medium: 70–92°, and Advanced: 92–110°; **S. Abduction ROM:** Low < 45°, Medium: 45–90°, and Advanced: 90–110°; **S. Medial Rotation ROM:** Low < 45°, Medium: 45–64°, and Advanced: 64–70°; **S. Lateral Rotation ROM:** Low < 20°, Medium: 20–40°, and Advanced: 40–60°; **Level of Exercise:** Beginner < 0.5; Medium = 0.5; Advanced > 0.5.

**Table 6:** Presents the results of the complete system, with shoulder joint motions and muscle strength as inputs and intensity of stretching and strengthening exercise as outputs.

	Shoulder Flexion ROM	Shoulder Abduction ROM	Shoulder Internal Rotation ROM	Shoulder External Rotation ROM	Strength (Percentage)	Output Strength (Percentage)	Output Stretch
Case 1	38	29	35	10	30	0	0.2781
Case 2	55	40	47	22	85	0	0.2454
Case 3	71	34	50	25	60	0	0.2474
Case 4	55	47	51	23	70	0	0.2928
Case 5	88	59	62	37	50	0	0.2781
Case 6	73	46	40	15	25	0	0.2781
Case 7	93	78	70	41	70	0	0.2735
Case 8	95	88	81	58	42	40	0.2905
Case 9	97	91	85	61	88	84	0.7163
Case 10	108	108	90	75	92	95.1905	0.7328
Case 11	130	180	100	80	100	0	0.7595
Case 12	100	95	40	15	60	0	0.2474
Case 13	60	100	65	50	75	0	0.2356
Case 14	105	40	68	53	90	0	0.2814
Case 15	85	105	70	55	30	0	0.2781

**Input Strength**—measures the strength of the patient’s impaired side with respect to their healthy side, represented by the percentage of MVC. 0–50%: Weak Strength, 50–80%: Moderate Strength, 80–100%: Substantial Strength. **Output Strength**—same range definition as input, only assigned when appropriate. **Output Stretch**— represents the intensity of stretching exercises to be assigned, defined over 0 to 1. Easy Stretch: 0–0.3, Hard Stretch: 0.6–1.0.
